# The outcomes of internal iliac artery preservation during endovascular or open surgery treatment for aortoiliac aneurysms

**DOI:** 10.1590/1677-5449.200087

**Published:** 2020-12-11

**Authors:** Rafael de Athayde Soares, Marcelo Fernando Matielo, Francisco Cardoso Brochado, Amanda Thurler Palomo, Rodrigo Andrade Lourenço, Caroline Tanaka, Roberto Sacilotto

**Affiliations:** 1 Hospital do Servidor Público Estadual de São Paulo, Serviço de Cirurgia Vascular e Endovascular, São Paulo, SP, Brasil.

**Keywords:** endovascular surgery, aortic aneurysm, iliac aneurysm, aortoiliac open surgery, cirurgia endovascular, aneurisma aorto-ilíaco, artéria ilíaca interna, cirurgia aberta

## Abstract

**Background:**

Internal iliac artery (IIA) preservation continues to be a challenge during open surgery or endovascular repair of abdominal aortoiliac aneurysm (AAIA).

**Objectives:**

To determine the results in terms of survival and clinical outcomes in patients with aortoiliac aneurysms (AAIA) treated with endovascular (EV) or open surgical (OS) repair.

**Methods:**

This was a retrospective consecutive cohort study of patients with AAIA who underwent EV or OS repair.

**Results:**

Post-procedure hospitalization time and intensive care unit stay were both longer in the OS group than in the EV group (7.08 ± 3.5 days vs. 3.32 ± 2.3 days; p = 0.03; 3.35 ± 2.2 days vs. 1.2 ± 0.8 days; p = 0.02, respectively). There were two cases of bowel ischemia (4.7%; OS 8.3% and EV 3.2%; p = 0.48), two cases of buttock claudication (4.7%; OS 8.3% and EV 3.2%; p = 0.48), and one case of sexual dysfunction (2.3% OS), all of them in patients with bilateral occlusion of the internal iliac artery (five patients, 11.6%; p = 0.035). Overall survival at 720 days was 80.6% in the EV group and 66.7% in the OS group (p = 0.58).

**Conclusions:**

In the present study, OS and EV repair of aortoiliac aneurysms had similar overall survival and outcomes. Preservation of at least one internal iliac artery is associated with good results and no further complications.

## Resumo

**Contexto:**

A preservação de uma artéria ilíaca interna continua a ser um desafio terapêutico nos pacientes com aneurismas aorto-ilíacos submetidos tanto ao tratamento endovascular quanto a cirurgia aberta.

**Objetivos:**

Determinar os resultados da sobrevida e desfechos clínicos em pacientes com aneurismas aorto-ilíacos (AAIA) que recebem reparo endovascular (EV) ou cirúrgico aberto (CA).

**Métodos:**

Este foi um estudo de coorte consecutivo e retrospectivo de pacientes com AAIA submetidos a reparo EV ou CA.

**Resultados:**

Houve maior tempo de internação pós-procedimento e permanência na unidade de terapia intensiva no grupo CA comparado com o grupo EV (7,08±3,5 dias vs. 3,32±2,3 dias; p = 0,03; 3,35±2,2 dias vs. 1,2±0,8 dias; p = 0,02, respectivamente). Houve dois casos de isquemia intestinal (4,7%; CA 8,3% e EV 3,2%; p = 0,48), dois casos de claudicação das nádegas (4,7%; CA 8,3% e EV 3,2%; p = 0,48) e um caso de disfunção sexual (2,3% CA), todos em pacientes com oclusão bilateral da artéria ilíaca interna (AII) (cinco pacientes, 11,6%; p = 0,035). A sobrevida global aos 720 dias foi de 80,6% no grupo EV e de 66,7% no grupo CA (p = 0,58).

**Conclusões:**

No presente estudo, o EV e o CA para aneurismas aorto-ilíacos apresentaram sobrevida e desfechos clínicos semelhantes. A preservação de pelo menos uma AII está associada a bons resultados e sem complicações adicionais.

## INTRODUCTION

Internal iliac artery (IIA) preservation continues to be a challenge during open surgery or endovascular repair of abdominal aortoiliac aneurysm (AAIA). Because the IIA is primarily responsible for pelvic perfusion, its preservation is important to avoid colonic ischemia, spinal ischemia, and buttock claudication and sexual dysfunction. Iliac aneurysms are associated with abdominal aortic aneurysms (AAA) in 20% to 30% of cases.^1^

Over the years, several treatment options have been developed to preserve the IIA. Open surgical (OS) approaches include bypass and even endoaneurysmorrhaphy. Endovascular techniques include covered stenting with sandwich techniques and, more recently, use of iliac branch devices.^2^^,^^3^ Nevertheless, preservation of at least one IIA is associated with safe and acceptable results without the need to preserve both IIAs, which can increase the degree of technical difficulty during surgery and the rate of complications, especially in patients with high cardiac risk.

Therefore, the objective of this study was to determine the outcomes of survival, endoleaks, reinterventions, buttock claudication, and perioperative mortality rate (PMR) in patients with AAIA treated with endovascular or OS repair related to IIA preservation.

## METHODS

Written patient informed consent was obtained for the study using a Helsinki Declaration compliant form. The study was approved by the research ethics committee. This was a retrospective, consecutive cohort study of patients with AAIA who underwent endovascular treatment or open surgical repair at the Division of Vascular and Endovascular Surgery, between January 2010 and April 2018. Patient data were collected from the vascular surgery service database and hospital records. Study was approved by Ethical Committee (number 34953614.3.0000.5463).

The study subjects were patients with AAIA who had undergone OS or endovascular repair. The decision on which type of surgical repair to use, OS or endovascular surgery, was made by the service’s vascular surgeons at weekly clinical discussion meetings. Patients with suitable aortic anatomy and high cardiac risk were scheduled to undergo endovascular repair. Patients with better cardiac risk and unsuitable anatomy for endovascular repair were scheduled for OS repair. All patients were evaluated preoperatively by the same cardiologist, with surgical risk stratification performed in accordance with the Brazilian Society of Cardiology’s preoperative evaluation guidelines. We defined the following three categories of cardiovascular mortality risk: low risk (<3%), moderate risk (3-15%), and high risk (>15%). Intraoperative arteriography procedures were re-evaluated to confirm that our departmental protocols had been executed accurately and to note the diameters and lengths of endoprostheses and stents, or coils. The indication for aneurysm surgery was either an AAA diameter larger than 55 mm or a common iliac artery diameter larger than 30 mm.

Endovascular repair was primarily performed using an aortoiliac endoprosthesis combined with IIA coil embolization. If both IIAs were involved and it was necessary to embolize both of them, a period of at least 2 weeks was observed between procedures. The preferred technique for embolizing the IIA was with proximal coil embolization, avoiding embolizing the more distal vessels. This technique is associated with fewer ischemic complications. More recently, depending on the availability of endoprostheses, the Zenith Bifurcated Iliac Side Branch Device (ZBIS®, Cook Medical) endoprosthesis was adopted in the vascular surgery department to preserve at least one IIA in conditions involving aortoiliac aneurysms. All patients were transferred to the intensive care unit (ICU) after surgery, where they remained for at least 24 hours before being transferred to a hospital unit.

The OS repairs were performed with a transperitoneal or retroperitoneal approach, depending on the vascular surgeon scheduled to treat the patient. The IIA aneurysms were preferably treated with artery ligation to exclude the IIA or, in some cases, the IIA was excluded with an endosuture in the proximal stump of the artery.

All of the patients were scheduled for follow-up at the hospital at 1, 6, and 12 months after discharge. After the first year, the patients were followed-up every 6 months and then every 12 months after the second year, at which time the following clinical criteria were evaluated: clinical examination, computed tomography scan, and Doppler ultrasonography. Technical success rates and early or late complications were reported according to the reporting standards of the Ad Hoc Committee for Standardized Reporting Practices in Vascular Surgery/International Society for Cardiovascular Surgery.^4^^,^^5^

Statistical analyses were performed using SPSS 15.0 for Windows®. Frequencies and descriptive statistics were analyzed. The chi-square test and Student’s *t* test were used to compare univariate analysis data. Survival curves were constructed using the Kaplan-Meier method to estimate limb salvage and survival rates. A p value < 0.05 was considered statistically significant. The Mann-Whitney and Wilcoxon tests were used as non-parametric tests. Analyses were performed within 720 days of the procedure.

### Surgical technique

End-to-end anastomosis was performed with a 16:8 or 18:9 Dacron Y graft between the proximal graft and the distal abdominal aorta, whereas the distal anastomosis was performed with end-to-end anastomoses in distal external iliac arteries, followed by ligation of the internal iliac arteries or end-to-end anastomoses at the iliac bifurcation, to preserve IIA perfusion. Endovascular procedures for internal iliac arteries consisted of the use of coil embolization for IIA exclusion, or use of branched iliac stent-grafts such as the ZBIS® to preserve IIA flow.

## RESULTS

A total of 43 patients were treated with OS or endovascular surgery between January 2010 and April 2018. The mean clinical follow-up period was 760 ± 80 days. Statistical analyses were performed at 720 days. Thirty-one of the 43 patients (72.1%) were scheduled to undergo endovascular surgery and 12 (27.9%) were treated with OS. Clinical characteristics were similar between the two groups, except for a higher prevalence of chronic kidney disease in the OS group (p = 0.04). The mean age of the whole cohort was 73.79 years, and most of patients were men (90.7%). The most prevalent disease in the whole cohort was hypertension (79.1%), followed by chronic kidney disease (27.9%). All the data are summarized in Table 1. Of the patient cohort, 46.5% had high cardiac risk, due to severe cardiac disease, and this risk was higher in the endo group than the OS group (p = 0.03).

**Table 1:** Clinical characteristics of patients.

**Variable**	**Total (n = 43)**	**Endo group**	**Open surgery group**	**p-value**
		**(n = 31, 72.7%)**	**(n = 12, 27.9%)**	
Age, years	73.79± 10.84	72.85 ± 8.3	73.48 ± 7.2	0.73
Males	39 (90.7%)	27 (87.1%)	12 (100%)	0.25
Hypertension	34 (79.1%)	23 (74.2%)	11 (91.7%)	0.16
Diabetes	6 (14%)	5 (16.1%)	1 (8.3%)	0.33
Ischemic heart disease	8 (18.6%)	6 (19.4%)	2 (16.7%)	0.27
Chronic kidney disease	12 (27.9%)	6 (19.4%)	6 (50%)	0.04
Chronic pulmonary disease	11 (25.6%)	9 (29%)	2 (16.7%)	0.23
Tobacco use	11 (25.6%)	8 (25.8%)	3 (25%)	0.30
Claudication history	4 (9.3%)	3 (9.7%)	1 (8.3%)	0.43
Increased cardiac risk	20 (46.5%)	17 (54.8%)	3 (25%)	0.03

Regarding the indications for aneurysm repair, most of them were related to aneurysm diameter (74.4%; endo group 74.2% and OS group 75%; p = 0.64), and 25.6% were related to symptomatic aneurysms (endo group 25.8%, OS group 25%; p = 0.64). Most of the symptomatic indications for repair were related to aneurysm expansion (72.7%); there were two cases of aneurysm rupture (one case in each group), and one case of blue toe syndrome (endo group). With regard to types of aneurysm, most of the patients had aortoiliac aneurysms (79.1%), followed by common iliac artery (9.3%), common and IIA (7%), and IIA (2.3%). These data are summarized in Table 2.

**Table 2:** Procedure data.

**Variable**	**Total (n = 43)**	**Endo group**	**Open surgery group**	**p-value**
		**(n = 31, 72.7%)**	**(n = 12, 27.9%)**	
Indication for surgery				
Aneurysm diameter	32 (74.4%)	23 (74.2)	9 (75%)	0.64
Symptomatic aneurysm	11 (25.6%)	8 (25.8%)	3 (25%)	0.64
Expansion	8 (18.6%)	6 (19.4%)	2 (16.7%)	0.54
Aneurysm rupture	2 (4.7%)	1 (3.2%)	1 (8.3%)	0.54
Blue toe syndrome	1 (2.3%)	1 (3.2%)	0 (0%)	0.54
Types of aneurysms				
Aortoiliac	34 (79.1%)	24 (77.4%)	10 (83.3%)	0.24
Common iliac artery	4 (9.3%)	4 (12.9%)	0 (0%)	0.02
Aortic aneurysm	1 (2.3%)	1 (3.2%)	0 (0%)	0.08
Internal iliac aneurysm	1 (2.3%)	1 (3.2%)	0 (0%)	0.08
Common iliac + internal iliac aneurysm	3 (7%)	1 (3.2%)	2 (16.7%)	0.08

In the endo group, most of the patients had endovascular repair of the aneurysm with an endoprosthesis with concomitant IIA embolization with coils (27 patients; 84.4%). Five patients received a ZBIS® device (Cook Medical). These patients were evaluated during follow-up and had no complications, with an IIA patency rate of 100% at 720 days, and no cases of endoleaks. The most used types of prostheses were Endurant Medtronic (15 cases; 48.4%), Zenith Cook (10 cases, 32.2%), and Gore Excluder (6 cases, 19.4%).

In the OS group, most of the patients underwent exclusion of the IIA through vessel ligation (10 patients). The other two patients had end-to-end anastomoses at the iliac bifurcation. Regarding patency of the IIA, before surgery, 95.3% of the patients had bilateral patency of the IIA; after surgery, 86% had at least one patent IIA. The mean diameters of the aortoiliac system were as follows: length of aortic neck 32.52 mm, diameter of aortic aneurysm 56.66 mm, right common iliac aneurysm 36 mm, left common iliac aneurysm 27.03 mm, right external iliac diameter 11.40 mm, left external iliac diameter 10.91 mm, right internal iliac diameter 16.77 mm, and left internal iliac diameter 14.50 mm (Table 3).

**Table 3:** Technical data on procedures.

**Variable**	**Total (n = 43)**	**Endo group**	**Open surgery group**	**p-value**
		**(n = 31, 72.7%)**	**(n = 12, 27.9%)**	
Endovascular procedures				
IIA coil embolization	27 (62.7)	27 (84.4)	0	
ZBIS®	5 (11.6)	5 (16.1%)	0	
Open surgery				
End-to-end anastomoses at iliac bifurcation	2 (4.65)	0	2 (16.6)	
Exclusion of the IIA	10 (23.2)	0	10 (83.3)	
Diameters (mm)				
Aortic aneurysm	56.66 ± 25.6	58.70 ± 26.8	59.94 ± 24.5	0.24
Right Common iliac aneurysm	36 ± 10.1	38 ± 11.2	34 ± 12.5	0.36
Left common iliac aneurysm	27.03 ± 11.3	26.09 ± 12.3	28.02 ± 14.3	0.45
Right external iliac	11.40 ± 0.8	10.80 ± 0.9	12.45 ± 0.7	0.90
Left external iliac	10.91 ± 0.7	10.83 ± 0.6	11.84 ± 0.8	0.87
Right external iliac	11.40 ± 0.9	10.87 ± 0.8	11.80 ± 0.7	0.97
Right internal iliac	16.77 ± 10.2	16.32 ± 14.3	16.28 ± 15.2	0.88
Left internal iliac	14.50 ± 15.6	14.28 ± 14.3	14.78 ± 15.6	0.76

Regarding complications, there were two cases of bowel ischemia (4.7%; endo group 3.2% and OS group 8.3%; p = 0.48), two cases of buttock claudication (4.6%; endo group 3.2% and OS group 8.3%; p = 0.48), and one case of sexual dysfunction (2.3%, in OS group). All of the patients with these complications had bilateral occlusion of the IIA (five cases, 11.6%; p = 0.035). The perioperative mortality rate was 11.6% (five cases overall; four cases in the endo group and one case in the OS group, p = 0.39). The 13% perioperative mortality in the endo group comprised 1 death related to bowel ischemia and 3 deaths related to cardiac ischemia complications. There were seven cases of immediate endoleak in the endo group (22.58%): two cases of endoleak type IA, four cases of endoleak type II, and one case of endoleak type IB. There were nine cases of late endoleaks: seven cases of endoleak type II and two cases of IB endoleak. The rate of freedom from reintervention was 73.3% in the endo group. Regarding the reinterventions, there were six cases of coil embolization in the IIA combined with glue embolization with Glubran®, and three cases of extension of the limb prosthesis to the external iliac artery combined with coil embolization of the IIA. In the OS group, there was one case of acute bowel ischemia due to internal iliac ligation during surgery, which caused the patient’s death; two cases of acute kidney dysfunction; and one case of bronchopneumonia. There was a longer post-procedure hospital stay in the OS group compared with the endovascular surgery group (7.08 ± 3.5 days vs. 3.32 ± 2.3 days; p = 0.03). Furthermore, the ICU stay was also longer in the OS group (3.35 ± 2.2 days vs. 1.2 ± 0.8 days; p = 0.02).

We performed univariate and multivariate Cox regression analyses to identify factors related to survival rate (Table 4). The Cox regression analysis for survival rates showed that elevated cardiac risk was related to poor survival rates (p = 0.001; hazard ratio [HR] = 1.40) in both univariate and multivariate analysis.

**Table 4:** Univariate and multivariate Cox regression analysis to identify factors related to survival rate.

**Variable**	**Univariate analysis**				**Multivariate analysis**			
	**B**	**OR**	**95% CI**	**p-value**	**B**	**OR**	**95% CI**	**p-value**
Cardiac disease	0.517	6.890	0.128-2.767	0.068	.542	7.890	0.226-10.567	0.880
Chronic kidney disease	0.299	0.830	0.262-2.501	0.547	1.286	1.412	0.820-4.294	0.642
Tobacco use	0.984	0.869	0.222-1.511	0.864	0.885	0.906	0.411-14.234	0.831
Diabetes	0.686	0.913	0.540-1.543	0.172	0.711	0.915	0.358-11.240	0.439
Indication for surgery	13.953	0.731	0.331-1.429	0.649	1.032	0.514	0.231-5.433	0.552
Bifurcated iliac endoprosthesis	0.453	0.806	0.231-0.811	0.615	0.249	0.262	0.612-1.811	0.618
Increased cardiac risk	12.564	1.499	1.456-2.365	0.001	11.595	1.500	1.234-4.321	0.002

B = coefficient; OR = odds ratio; CI = confidence interval.

The univariate and multivariate Cox regression analysis related to reintervention rates showed that late endoleak was the only factor related to reintervention (HR = 3.7; p = 0.035) (Table 5).

**Table 5:** Univariate and multivariate Cox regression analyses to identify factors related to reintervention.

**Variable**	**Univariate analysis**				**Multivariate analysis**			
	**B**	**OR**	**95% CI**	**P**	**B**	**OR**	**95% CI**	**P**
Cardiac disease	.615	6.000	.028-9.790	.057	.597	7.000	0.496-8.954	.780
Chronic kidney disease	.897	7.637	0.163-4.401	.437	0.386	2.315	1.720-3.394	.432
Tobacco use	.984	0.869	0.222-1.511	.864	0.885	0.906	0.411-14.234	.831
Diabetes	.686	0.913	0.540-1.543	.172	0.711	0.915	0.358-11.240	.439
Indication for surgery	10.953	0.731	0.431-2.429	.529	2.132	0.347	0.821-4.363	.890
Bifurcated iliac endoprosthesis	1.453	0.806	1.831-6.911	.415	0.589	0.876	0.712-2.815	.578
Immediate endoleak	8.554	1.359	0.356-1.265	.987	2.395	2.308	2.564-4.121	.876
Late endoleak	5.564	3.700	1.321-4.875	.035	4.765	3.400	1.542-4.589	.035
Number of patent internal iliac arteries	0.709	1.954	0.062-3.888	.501	0.709	1.054	0.098-2.768	.890

B = coefficient; OR = odds ratio; CI = confidence interval.

**Figure 1:**
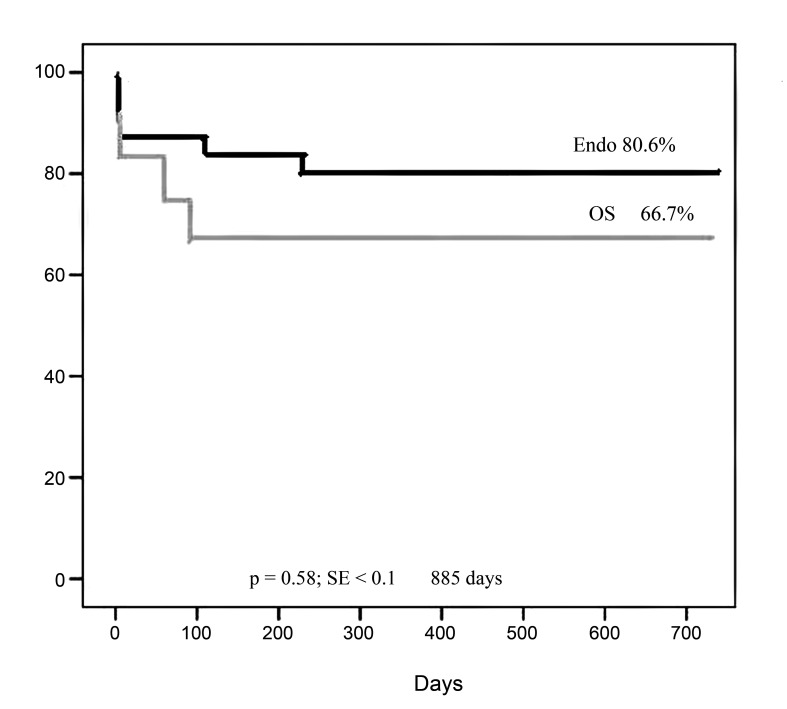
Overall survival at 720 days by the Kaplan-Meier method was 80.6% in the endo group and 66.7% in the open surgery (OS) group, but there was no statistically significant difference between the groups (p = 0.58).

Overall survival rates at 720 days according to the Kaplan-Meier method were 80.6% in the endo group and 66.7% in the OS group, but with no statistically significant difference between the groups (p = 0.58) (Figure 1).

## DISCUSSION

According to American Society for Vascular Surgery documents, preservation of at least one IIA is strongly recommended during repair of aortoiliac aneurysms. Furthermore, it is also recommended that bilateral IIA occlusion be conducted in two stages, separated by at least 1 to 2 weeks, if required for endovascular correction of aortic aneurysm.^6^ Our vascular department’s practice is to strictly adhere to this recommendation during correction of abdominal aortoiliac aneurysms. Regarding patency of the IIA, 95.3% of the patients had bilateral IIA patency before surgery. After surgery, 86% had at least one patent IIA. All patients in this cohort who had bilateral IIA occlusion had complications afterwards: two patients with bowel ischemia (4.7%; endo group 3.2% and OS group 8.3%; p = 0.48), two patients with buttock claudication (4.7%; endo group 3.2% and OS group 8.3%; p = 0.48), and one patient with sexual dysfunction (2.3%, OS group). This therefore underscores the need to preserve at least one IIA to avoid ischemic complications.

Bosanquet et al.^7^ published a systematic review in which 2671 patients and 2748 IIAs were analyzed. Buttock claudication occurred in 27.9% of patients, although in 48.0% it resolved after 21.8 months. Buttock claudication rates were 32.6% with coils, 23.8% with plugs, and 12.9% with coverage alone, and fewer with unilateral (vs. bilateral) IIA treatment (OR 0.57, 95% CI 0.36-0.91). There were two cases of buttock claudication (4.65%) in the present cohort. Buttock claudication was related to bilateral IIA exclusion, while patients who had unilateral IIA exclusion did not have buttock claudication. IIA embolization was performed using coils in 100% of cases in the present cohort. These findings are similar to those that Mansour et al.^8^ reported in a retrospective study in which buttock claudication and sexual dysfunction rates were significantly higher in the group that underwent bilateral IIA occlusion than in the group with at least one IIA preserved (p < 0.05). Their conclusion was that at least one IIA should be salvaged in cases of bilateral involvement.

Another important embolization technique for avoiding ischemic complications that is employed at our vascular department is proximal IIA embolization, avoiding embolizing the more distal vessels, such as the gluteal branches. Maleux et al.^9^ have concluded that ipsilateral coil or microcoil embolization of the proximal IIA before stent-graft extension in patients previously treated by an aortic stent-graft seems to be safe and feasible, with favorable outcomes after a mean follow-up period of 39 months. The incidence of buttock claudication was 38% of patients and there were no type II endoleaks through the coil-embolized internal iliac arteries. According to Bannazadeh et al.,^10^ in a retrospective review of all patients who underwent elective endovascular aneurysm repair (EVAR), there were no significant differences in reintervention rates between iliac limb extension into the external iliac artery with IIA coil embolization, flared iliac limb 20 mm or greater in diameter to the iliac bifurcation, or iliac limb 20 mm or less ending proximal to the concomitant common iliac artery aneurysm (4.5% vs. 4.8% vs. 6.2%; P = 0.802) over a mean 59.8 months of follow-up.

In the present study, five patients were treated using a ZBIS® device (ZBIS®, Cook Medical). These patients were evaluated and the procedure was associated with high technical success rates and no cases of IIA occlusion during follow-up or immediate or late endoleaks. This is comparable to what Delay et al.^11^ found with relation to short and mid-term results for the ZBIS® device. They found that primary patency of the internal iliac side branch was 84% at 1 year and 76% at 2 years (five perioperative IIA occlusions and one late occlusion). Freedom from reintervention was 89% at 1 and 2 years. In the present cohort, IIA branch patency was achieved in 100% of cases, but few cases were treated and follow-up was only 2 years. Farivar et al.^12^ found freedom from type I or III endoleaks at 3, 5, and 10 years in 99% of the cases in which a bifurcated iliac endoprosthesis was used to treat aortoiliac aneurysms and reported primary patency at 3, 5, and 10 years of 94%, 94%, and 77%, respectively.

In a very recent publication, Mendes et al.^13^ evaluated the perioperative outcomes of patients with iliac aneurysms treated by OS versus endovascular repair with iliac branch endoprostheses. They found that perioperative mortality occurred in one patient in the OS group (4%), with no mortality in the iliac branch endoprosthesis group (P = 0.37). Furthermore, the total length of hospital and ICU stay was longer for the OS group compared with the iliac branch endoprosthesis group (total stay 7.5 ± 3.4 vs. 1.7 ± 1.4 days for IBE, P < 0.0001, and ICU 3.3 ± 2.1 vs. 0.1 ± 0.4 days, P < 0.0001). These data are similar to what we found in the present cohort, in which lengths of both hospital and ICU stays were longer in the OS group. This may be because of the laparotomy and more invasive procedure performed in OS than in endo repair. Although not statistically significant, in this study there were more cases of perioperative deaths in the endovascular group than in the OS group and this result was because of the higher cardiac risk in the endo group compared with the OS group, with statistical significance.

Kobe et al.^14^ found 17 endoleaks (6 type I, 10 type II, and 1 type III) in a group of 72 patients with 85 IIAAs treated with endovascular repair. The overall reintervention rate was 16.7%. The primary patency rate was 98.6%. In the present study, we found primary IIA patency of 86% at 720 days, and a reintervention rate of 26.4% at 720 days. The main factor related to reintervention in the present cohort was late endoleak, and the endovascular technique performed did not influence the rates of endoleak or reintervention. The overall survival at 720 days was similar in the OS and endo groups in the present cohort and is similar to rates found by the United Kingdom EVAR Trial Investigators, where the endovascular repair group had an early benefit with respect to aneurysm-related mortality, but the benefit was lost by the end of the study, at least partially because of fatal endograft ruptures (adjusted HR, 0.92; 95% confidence interval [CI], 0.57-1.49; P=0.73).^15^

This study has some limitations, in that it is a single-center retrospective study with a cohort that is not very large, especially in terms of the patients who underwent repair with iliac branch endoprostheses and the conclusions are based on a small number of operations from a retrospective study. Larger, prospective studies should be conducted.

## CONCLUSION

In the present study, the OS and endovascular procedures for aortoiliac aneurysms had similar overall survival rates, buttock claudication rates, and perioperative mortality rates. Preservation of at least one IIA is associated with good results and no further complications. Reinterventions were performed exclusively in the endo group, due to late endoleaks. However, the endo group had shorter hospital and ICU stays compared with the OS group.
